# CDSnake: Snakemake pipeline for retrieval of annotated OTUs from paired-end reads using CD-HIT utilities

**DOI:** 10.1186/s12859-020-03591-6

**Published:** 2020-07-24

**Authors:** Yulia Kondratenko, Anton Korobeynikov, Alla Lapidus

**Affiliations:** 1 Center for Algorithmic Biotechnology, Institute for Translational Biomedicine, St. Petersburg State University, St. Petersburg, Russia 199004; 2 Department of Statistical Modelling, St. Petersburg State University, St. Petersburg, Russia 198515

**Keywords:** 16S metagenomics, Operational taxonomic units, Pipeline

## Abstract

**Background:**

Illumina paired-end reads are often used for 16S analysis in metagenomic studies. Since DNA fragment size is usually smaller than the sum of lengths of paired reads, reads can be merged for downstream analysis. In spite of development of several tools for merging of paired-end reads, poor quality at the 3′ ends within the overlapping region prevents the accurate combining of significant portion of read pairs. Recently CD-HIT-OTU-Miseq was presented as a new approach for 16S analysis using the paired-end reads, it completely avoids the reads merging process due to separate clustering of paired reads. CD-HIT-OTU-Miseq is a set of tools which are supposed to be successively launched by auxiliary shell scripts. This launch mode is not suitable for processing of big amounts of data generated in modern omics experiments. To solve this issue we created CDSnake – Snakemake pipeline utilizing CD-HIT tools for easier consecutive launch of CD-HIT-OTU-Miseq tools for complete processing of paired end reads in metagenomic studies. Usage of pipeline make 16S analysis easier due to one-command launch and helps to yield reproducible results.

**Results:**

We benchmarked our pipeline against two commonly used pipelines for OTU retrieval, incorporated into popular workflow for microbiome analysis, QIIME2 - DADA2 and deblur. Three mock datasets having highly overlapping paired-end 2 × 250 bp reads were used for benchmarking - Balanced, HMP, and Extreme. CDSnake outputted less OTUs than DADA2 and deblur. However, on Balanced and HMP datasets number of OTUs outputted by CDSnake was closer to real number of strains which were used for mock community generation, than those outputted by DADA2 and deblur. Though generally slower than other pipelines, CDSnake outputted higher total counts, preserving more information from raw data. Inheriting this properties from original CD-HIT-OTU-MiSeq utilities, CDSnake made their usage handier due to simple scalability, easier automated runs and other Snakemake benefits.

**Conclusions:**

We developed Snakemake pipeline for OTU-MiSeq utilities, which simplified and automated data analysis. Benchmarking showed that this approach is capable to outperform popular tools in certain conditions.

## Background

Sequencing of 16S rRNA or its fragments is a commonly used method for cost-efficient characterizing of microbial communities. Illumina paired-end reads are often used as sequencing method. Since even short variable regions of 16S provide sufficient information for microbe identification, sequenced fragment length is often taken smaller than the sum of lengths of paired reads. Thus reads of pairs can be merged for downstream analysis, which commonly includes clustering of sequences and matching the resulting clusters’ representative sequences with annotated database. In spite of the development of several tools for merging of paired-end reads [1, 2], poor quality sequences at the 3′ ends of both paired-end reads in the overlapping region prevent the correct assembly of significant portion of read pairs. Incorrectly or uncertainly merged reads either have to be excluded from downstream analysis or retained with high risk of spurious sequences creation.

Recently CD-HIT-OTU-Miseq [3] was presented as a new approach, entirely avoiding reads merging due to separate clustering of paired reads and discarding of reads voting for non-matching clusters as chimeric. We considered that this approach could improve important step of OTU table generation, by discarding smaller portion of reads in process of OTU retrieval. Thus larger portion of source information will be saved for downstream analysis, and more profound understanding of explored community structure can be achieved. CD-HIT-OTU-Miseq utilities are command line tools written in C++ and Perl. Here we combined CD-HIT-OTU-Miseq utilities into pipeline using Snakemake [4] workflow. Snakemake is fully portable, as only a Python installation is required to run Snakefiles, and does not require tight integration of tools into the workflow system. It provides automatic scalability because it optimizes the number of parallel processes with respect to provided CPU cores and needed threads and can make use of single machines as well as cluster engines without modifying the workflow. Usage of Snakemake makes application of CD-HIT tools easier due to one command launch of pipeline and provides better reproducibility of the results. Snakemake also allows to resume interrupted work and reports percentage of accomplished tasks, making large-scale data processing handier.

## Implementation

Pipeline takes as input a folder with paired-end reads in form of fastq files and database of annotated microbial 16S sequences (for instance, Greengenes [5] or SILVA [6]) in form of fasta files. If quality of reads was preliminarily assessed, the lengths of “good” parts of R1 and R2 reads can be provided as input parameters. Parameters of “good” parts depend on overall quality profile of reads and their number, but generally quality should exceed 25 with no sudden drops. Corresponding parts of reads are considered for sequences clustering. Default lengths of “good” parts for R1 and R2 are set to 200 and 180 bp correspondingly. Reads are then put into separate folders for each pairs, and sample file including names of reads for all pairs is created. 16S-ref-db-PE-splice.pl utility then used to cut database 16S sequences into fragments corresponding in lengths and quantities to randomly selected sample. Reads sequences are then filtered by quality using Trimmomatic [7]. Filtered sequences are clustered at 99% using cd-hit-est utility to discard chimeric reads. The important feature of CD-HIT-OTU-MiSeq is that R1 reads of pairs are clustered together, separate form R2 reads, and then clusterization with same parameters is made for R2 reads. Thus reads of pairs do not need to be merged or concatenated. Chimeric reads detection is possible when reads from singe pair vote for non-matching clusters of two clusterizations (and these clusters are large enough). Remaining reads and fragments from annotated database are then clustered at 97% similarity to yields annotated clusters commonly named Operational Taxonomic Units, or OTU. 16S reads clustered with 97% similarity result in read groups corresponding to species or close taxonomic levels. Clusters that matched with some sequences from annotated database receive the annotation written into output OTU file.

## Results

We benchmarked our pipeline against two commonly used pipelines for OTU retrieval, incorporated into popular workflow for microbiome analysis, QIIME2 [8]. These pipelines are DADA2 [9] and deblur [10].

DADA2 iteratively divides reads into groups until each group is highly likely originates from central sequence, according to error model for Illumina amplicon reads. This central sequence is supposed to represent the original genotype, which might be sequenced with some errors.

Deblur modifies abundances of reads using Hamming distances and subtracting abundances of reads which are considered to be erroneous version of given read from this read abundance. Reads which abundances drops to zero are discarded. After that UCHIME [11] algorithm as implemented by VSEARCH [12] is used for chimeras filtering.

Merging of paired-end reads stage is incorporated in DADA2 pipeline. Since deblur doesn’t work with paired-end reads (and processes only R1 reads if paired-end reads are provided), reads of pairs should be merged prior to running deblur. We used VSEARCH incorporated in QIIME workflow in “join-pairs” mode to merge reads before deblur usage.

Three mock datasets having highly overlapping paired-end 2 × 250 bp reads were used for benchmarking - Balanced [13], HMP [14], and Extreme [9]. The Balanced community contained 57 bacteria and archaea at nominally equal frequencies, the HMP community contained 21 bacteria at nominally equal frequencies, and the Extreme community contained 27 bacterial strains at frequencies spanning five orders of magnitude and differing over the sequenced region by as little as 1 nucleotide (nt). Balanced dataset had higher sequence quality (Mean Q = 35.9 forward/33.5 reverse); Extreme had moderate quality (33.0/29.3); and HMP had lower quality (32.3/28.7). We also used set of all 3 mock datasets (further referred as “All”) to create input data with 3 samples of varying quality, so each mock dataset there represented separate sample.

Benchmarking results are presented in Table 1. As expected, CDSnake outputted less OTUs than DADA2 and deblur, since last two tools aim to output sub-OTUs by error correction, and OTU-MiSeq doesn’t process errors and tries to output most correct OTUs using clustering [1]. The exception was HMP dataset of lowest quality, where deblur outputted less OTUs than CDSnake (53 vs. 59). In this level of errors in input data clustering of reads by CD-HIT utilities outputted more OTUs than deblur after his dropping of erroneous sequences. However, on Balanced and All datasets number of OTUs outputted by CDSnake was closer to real number of strains which were used for mock community generation, than those outputted by DADA2 and deblur. On Extreme dataset CDSnake, as expected, performed worse than DADA2 and deblur, since clustering algorithm cannot separate sequencing errors from actual 1-nt differences, present between strains in this community.

**Table 1:** Benchmarking of DADA2, deblur and CDSnake on three mock community datasets and set of these datasets, where each mock dataset represented one sample (All row)

	actual number of microbial strains	pipeline	OTUs/Features outputted	OTUs/Features annotated	OTUs/Features correctly annotated	Microorganisms correctly discovered	Total count
Balanced	59	dada2	91	**67**	**57**	39	29,344
		deblur	343	280	244	**41**	200,317
		CDSnake	**55**	45	31	29	**524,604**
HMP	21	dada2	68	49	31	**19**	186,027
		deblur	**53**	36	**23**	18	175,027
		CDSnake	59	**21**	17	17	**214,831**
Extreme	27	dada2	**30**	**26**	**25**	**18**	**1,371,591**
		deblur	18	15	14	13	775,144
		CDSnake	12	12	9	8	1,357,589
All	~ 105	dada2	180	**134**	**116**	**77**	1,392,444
		deblur	393	317	274	68	224,388
		CDSnake	**123**	31	26	25	**2,231,154**

Mechanisms of OTUs annotation differ in CD-HIT-OUT-MiSeq and QIIME2, in which we testes DADA2 and deblur. CD-HIT-OTU-MiSeq annotation output is binary – it either has annotation in any taxonomic level or outputs “None” in corresponding field. QIIME2 classifiers output some taxonomic annotation for each OTU and provide confidence scores for all annotations. We considered OTUs annotated for QIIME2 pipelines if confidence score exceeded 0.9.

Since we used mock datasets, correct annotations were known for each community. We considered feature correctly annotated if corresponding genus was present in published dataset content. Number of these features is presented in column “OTUs/Features correctly annotated” of Table 1. Notably, in some cases several features corresponded to single microorganism from source dataset. This could indicate that more genetic complexity was present in microorganism DNA which authors of source datasets used to create mock community and considered to belong to single strain. Otherwise, especially in cases when too many features were outputted for one source strain, this heterogeneity can be artefact of sequencing errors or incorrect work of error correction algorithms, if they were applied.

Considering such complex mapping of annotated features to known annotations, we also provide second measure of correctness of annotation – number of microorganisms form source community, which received annotation on genus level. Number of these microorganisms is presented in column “Microorganisms correctly discovered”.

Number of annotated OTUs was generally closer to true number of microbial species for DADA2 pipeline, and in one case, with HMP dataset, CDSnake outputted better result. Annotations were generally closer to expected ones for dada2 pipeline.

Total outputted microbial counts for mock datasets were higher for CDSnake than for DADA2 and deblur. Extreme dataset was an exception here since DADA2 outputted more counts than CDSnake in this case, though numbers didn’t differ substantially (1,371,591 for DADA2 vs. 1,357,589 foe CDSnake). Debur outputted the least counts in most of cases with exception of Balanced dataset, where DADA2 outputted least counts.

We benchmarked time of all runs on two cores of Asus Aspire S13 laptop. In addition to 3 pipelines, DADA2, deblur and CDSnake we added original CD-HIT-OTU-MiSeq utilities for speed benchmarking (we don’t provide data on original CD-HIT-OUT-MiSeq utilities in Table 1 since they were identical to results of CDSnake). Deblur was faster than other tools in all tested cases. On Extreme dataset CD-HIT-OUT-MiSeq utilities and CDSnake runs took significantly longer than DADA2 and deblur. For Balanced and HMP dataset running times were comparable with CDSnake running faster than DADA2 on HMP dataset and longer on Balanced dataset. Running time didn’t differ substantially between CD-HIT-OUT-MiSeq utilities and CDSnake but tend to take slightly longer for CDSnake. This can be explained by usage of additional python components which are necessary to run Snakemake pipelines.

## Conclusion

Microbiome research is a complex field with common trade-offs between quality and quantity of data that could be used for analysis. As shown in Tables 1 and 2, choice of tool for certain task should depend on most important parameters of output, such as number of OTUs or total count, as long as time limits. Quality of input data and complexity of studied microbial community also should be considered when tools are selected. CD-HIT-OTU-MiSeq provides one more approach for amplicon analysis capable to outperform popular tools in certain conditions. We developed Snakemake pipeline for OTU-MiSeq utilities, which can be helpful for easier automated runs.

**Table 2:** Time benchmarking of DADA2, deblur, source CH-HIT-OUT-Miseq utilities and CDSnake on three mock community datasets and set of these datasets (All row)

	Time, sec			
	DADA2	deblur	source CD-HIT	CDSnake
HMP	4614.8	**1371.72**	2149.59	2263.83
Balanced	8207.96	**2558.82**	9345.62	9563.57
Extreme	7362.38	**3599.73**	47,684.8	43,170.37
All	17,509.9	**6376.05**	39,775.51	39,891.53

## Data Availability

Pipeline code and manual are available at https://github.com/ydkondratenko/cdsnake. Data for benchmarking were drawn from cited papers.

## Abbreviation

OTU: Operational taxonomic units
