# Correction to: CDSnake: Snakemake pipeline for retrieval of annotated OTUs from paired-end reads using CD-HIT utilities

**DOI:** 10.1186/s12859-020-03709-w

**Published:** 2020-08-19

**Authors:** Yulia Kondratenko, Anton Korobeynikov, Alla Lapidus

**Affiliations:** 1 Center for Algorithmic Biotechnology, Institute for Translational Biomedicine, St. Petersburg State University, St. Petersburg, Russia 199004; 2 Department of Statistical Modelling, St. Petersburg State University, St. Petersburg, Russia 198515

**Correction to: BMC Bioinformatics 21, 303 (2020)**

**https://doi.org/10.1186/s12859-020-03591-6**

Following publication of the original article [1], the authors would like to correct the Funding note section:

The sentence currently reads: Scientific research performed at the Computing Center of Research park of St. Petersburg State University.

The sentence should read: Scientific research performed at the Computing Center of Research park of St. Petersburg State University. Development of the pipeline and publication of this supplement was funded by Russian Science Foundation grant #19–16-00049.
